# High throughput assessment of blueberry fruit internal bruising using deep learning models

**DOI:** 10.3389/fpls.2025.1575038

**Published:** 2025-05-21

**Authors:** Chenjiao Tan, Changying Li, Penelope Perkins-Veazie, Heeduk Oh, Rui Xu, Massimo Iorizzo

**Affiliations:** ^1^ Department of Agricultural and Biological Engineering, University of Florida, Gainesville, FL, United States; ^2^ Department of Horticultural Science, North Carolina State University, Raleigh, NC, United States

**Keywords:** segmentation, bruising ratio, YOLO, cultivars, firmness

## Abstract

The rising costs and labor shortages have sparked interest in machine harvesting of fresh-market blueberries. A major drawback of machine harvesting is the occurrence of internal bruising, as the fruit undergoes multiple mechanical impacts during this process. Evaluating fruit internal bruising manually is a tedious and time-consuming process. In this study, we leveraged deep learning models to rapidly quantify berry fruit internal bruising. Blueberries from 61 cultivars of soft to firm types were subjected to bruise over a three-year period from 2021-2023. Dropped berries were sliced in half along the equator and digitally photographed. The captured images were first analyzed using the YOLO detection model to identify and isolate individual fruits with bounding boxes. Then YOLO segmentation models were performed on each fruit to obtain the fruit cross-section area and the bruising area, respectively. Finally, the bruising ratio was calculated by dividing the predicted bruised area by the predicted cross-sectional area. The mean Average Precision (mAP) of the bruising segmentation model was 0.94. The correlation between the bruising ratio and ground truth was 0.69 with a mean absolute percentage error (MAPE) of 15.87%. Moreover, analysis of bruising ratios of different cultivars revealed significant variability in bruising susceptibility and the mean bruising ratio of 0.22 could be an index to differentiate the bruise-resistant and bruise-susceptible cultivars. Furthermore, the mean bruising ratio was negatively correlated with mechanical texture parameter, Young’s modulus 20% Burst Strain. Overall, this study presents an effective and efficient approach with a user-friendly interface to evaluate blueberry internal bruising using deep learning models, which could facilitate the breeding of blueberry genotypes optimized for machine harvesting. The models are available at https://huggingface.co/spaces/c-tan/blueberrybruisingdet.

## 1 Introduction

Blueberry (*Vaccinium corymbosum L.*) has surged in popularity in the past decade and the retail per capita availability increased from 1.2 pounds in 2011 to 2.3 pounds in 2021 (USDA-ERS, 2024). Blueberries contribute significant amounts of vitamins, minerals, dietary fiber, and anthocyanins (Golovinskaia and Wang, 2021) and blueberry consumption is associated with many health benefits (Stull et al., 2024). In the United States, blueberry production increased from 143 million kg in 2013 to 294 million kg of blueberries in 2023 (USDA-NASS, 2023). Although machine harvesting can improve harvest efficiency and labor productivity, fresh market blueberries are generally hand-harvested, due to bruising damage from machine harvesting operations (Gallardo et al., 2018). Recently, researchers improved the catch plates by installing soft catch surfaces on a catch frame to reduce the impact bruising (DeVetter et al., 2019; Sargent et al., 2021).

Developing suitable blueberry varieties for machine harvest and fresh market is also essential. Breeding cultivars with improved resistance to impact force and thus minimizing internal bruising, are particularly critical for successful mechanical harvesting. Bruising is a type of subcutaneous tissue failure without rupture of the skin, as the cell breakage is caused by mechanical impact, which leads to softer tissues in bruised fruit compared to healthy fruit (Hussein et al., 2018). Consequently, firmness may be useful as an indirect index for fruit bruising assessment. Firmness measurements have included texture analyzers, penetrometer or durometer tests, acoustic impulse response analysis, and vibration-based methods (Yu et al., 2014; Jiang et al., 2016), with varying success. For instance, FirmTech II and Penefel durometer performed better in soft fruit but not as well in moderate or firm fruit (Moggia et al., 2022). Recently, several studies have suggested that instruments that allow the measurement of multiple mechanical parameters, such as texture analyzers, can better dissect texture variation among blueberry genotypes and provide more robust data (Giongo et al., 2013, 2022; Mengist et al., 2024; Oh et al., 2024a; Oh et al., 2024b). Despite the advances in blueberry texture analysis, it is still unknown which mechanical texture components contribute to reduced internal bruising.

The discolored tissues of bruised fruit provide an obvious characteristic to differentiate healthy fruits and bruised fruits. While visual assessment is a simple way to evaluate fruit bruising, this method is time-consuming, laborious, and subjective. Additionally, for fruit with dark skin, such as blueberry, it is difficult to differentiate the discolored area externally. A typical assessment practice developed for blueberries is to slice blueberries to expose the internal tissues (Ni et al., 2022). Each blueberry is sliced along the equator and placed on a flat surface, then experienced evaluators immediately estimate the bruising ratio between the bruising area and the entire sliced surface area. As results are subjective and affected by the evaluator, sliced samples are photographed, and software is used to select the bruising area and sliced surface area on the image. The bruising ratio then can be calculated based on the pixel number of each selected area. Although this method is more accurate, it is much more time consuming than rough visual estimation and is destructive. Some researchers have leveraged techniques as non-destructive approaches for fruit bruising detection, including X-ray (Diels et al., 2017; Huang and Liang, 2024), magnetic resonance imaging (Razavi et al., 2018; Noshad et al., 2020), computed tomography (Azadbakht et al., 2019; Wood et al., 2024) and hyperspectral imaging (Fan et al., 2018; Fu and Wang, 2022; Okere et al., 2023; Bu et al., 2024; Liu et al., 2024). These techniques require expensive devices and a strict imaging environment. Additionally, data collection and analysis are very complex for breeders.

With the development of deep learning, many computer vision tasks, including classification, object detection, and segmentation can be solved by deep learning-based methods, especially conventional neural networks (CNNs). Several popular CNNs used for classification include VGGNet (Simonyan, 2014), ResNet (He et al., 2016), DenseNet (Huang et al., 2017), and EfficientNet (Tan and Le, 2019). The CNNs used for object detection can be categorized as two-stage models and single-stage models (Tan et al., 2024). The most well-known two-stage models are R-CNN series, including R-CNN, Fast R-CNN and Faster R-CNN. The region proposal network was used to predict the candidates of the objects and then the candidates were further refined to get the bounding boxes of target objects. The best-known one-stage model is the YOLO series for the balance between accuracy and inference speed. The latest version of YOLO series models is YOLO11, which used Feature Pyramid Network (FPN) and Path Aggregation Network (PAN) for feature extraction, and the bounding boxes of target objects were directed regressed. Subsequently, R-CNN and YOLO series were developed for object segmentation tasks with pixel-wise prediction to represent the target objects, named Mask R-CNN and YOLO-seg.

The aforementioned models have been widely employed in vegetable and fruit postharvest quality assessment due to their remarkable accuracy and adaptability. Faster R-CNN has been a popular choice for bruise detection, with several studies improving its architecture to enhance performance (Hou et al., 2024). Similarly, Mask R-CNN has also been proven effective, particularly for segmentation tasks, such as achieving 99.8% accuracy in olive segmentation (Macías-Macías et al., 2023).

YOLO models have gained widespread use for bruising detection due to their accuracy and speed (Tan et al., 2025). For instance, YOLOv4 was simplified to detect apple defects using channel and layer pruning methods, achieving an mAP of 93.74% with an inference time of 10.82 ms (Fan et al., 2022). Building on the success of YOLOv5, improvements were made for apple bruising detection on thermal images, resulting in an impressive mAP of 98.08% (Lin et al., 2023). Similarly, enhancements to YOLOv5s for kiwifruit defect detection demonstrated high detection accuracies across various defect categories, including 98.8% for healthy samples and 95.9% for sunburned samples (Wang et al., 2023). Another study also employed YOLOv5 to detect hidden bruises of kiwifruit on hyperspectral images, achieving an mAP of 99.12% (Bu et al., 2024). Additionally, YOLOv5 was also improved for defect detection of pears, obtaining an mAP of 0.939 and a detection speed of 2.2 ms (Wang et al., 2024). Moreover, a study compared Faster R-CNN, YOLOv3-Tiny, and YOLOv5s for apple bruising detection on near-infrared images, and the accuracy of the three algorithms was higher than 96% (Yuan et al., 2022). Similarly, several CNNs including YOLOv5, YOLOv8, Faster R-CNN, and Mask R-CNN were compared for African plum defect detection and YOLOv8 achieved the highest mAP of 93.6% (Fadja et al., 2024).

Although many studies have achieved promising results using deep learning methods for bruising detection, most have focused on individual fruit bruising detection and required a conditional environment for imaging, especially with hyperspectral cameras, limiting method application and efficiency. In our previous research, we developed a U-Net-based blueberry bruising detection method (Ni et al., 2022). Although the method achieved an mAP of 0.773 for bruising segmentation, this study used a limited dataset with 68 images, and all images were collected in the same year. With the recent development of deep learning, many new models have been proposed with higher accuracy. In this study, we explored the latest object detection and instance segmentation algorithms of the YOLO series for blueberry fruit detection and bruising segmentation. Additionally, over 40 blueberry cultivars were sampled and photographed for bruising analysis each year for 3 years. Utilizing a texture analyzer, a Young’s modulus parameter, Young’s modulus 20% Burst Strain (YM20_BrSt), of each cultivar was measured as an index of firmness, and the correlation between YM20_BrSt and bruising ratio was evaluated. The objectives of this paper were to: (1) build a blueberry impact bruising dataset, train and evaluate deep learning models for internal bruising ratio detection; (2) analyze the bruising ratio over three different years and 61 cultivars; (3) evaluate the correlation between firmness and bruising ratio and distinguish bruise-resistant and bruise-susceptible cultivars, and (4) develop a user-friendly interface for bruising detection and calculation.

## 2 Materials and methods

### 2.1 Overview of the method

**Figure 1:**
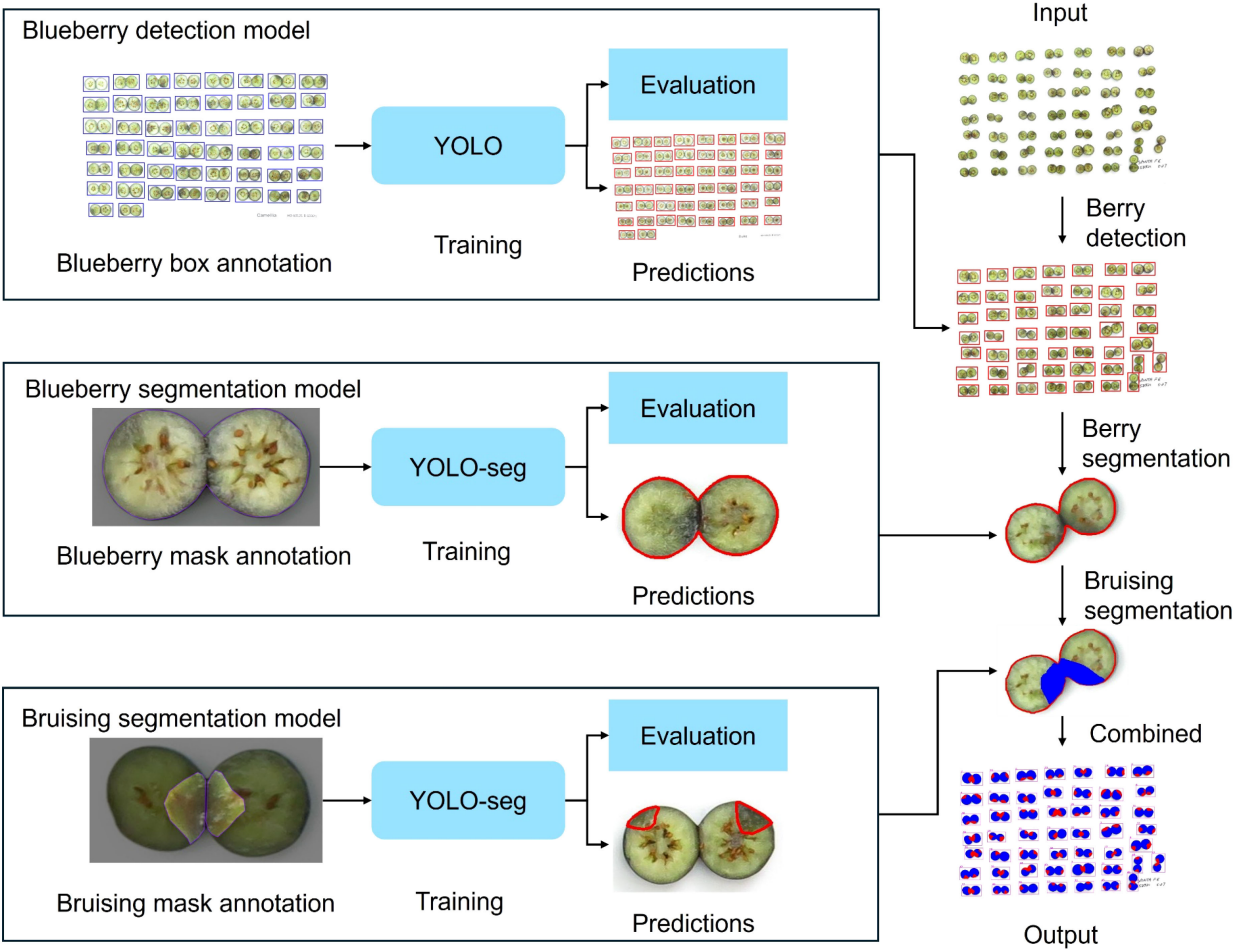
Overview of the developed approach. It consists of three YOLO-based models: (1) blueberry detection model, (2) blueberry segmentation model, and (3) blueberry internal bruising segmentation model.

The overview pipeline for the blueberry internal bruising detection is illustrated in **Figure 1**. It consists of three YOLO-based models: (1) blueberry detection model, which was used to detect the bounding box of each individual blueberry; (2) blueberry segmentation model, which was employed to segment the blueberry area and (3) bruising segmentation model, which was leveraged to segment the bruising area. After obtaining the blueberry and bruising area, the bruising ratio of each blueberry was then calculated based on the pixel number.

### 2.2 Data collection

**Figure 2:**
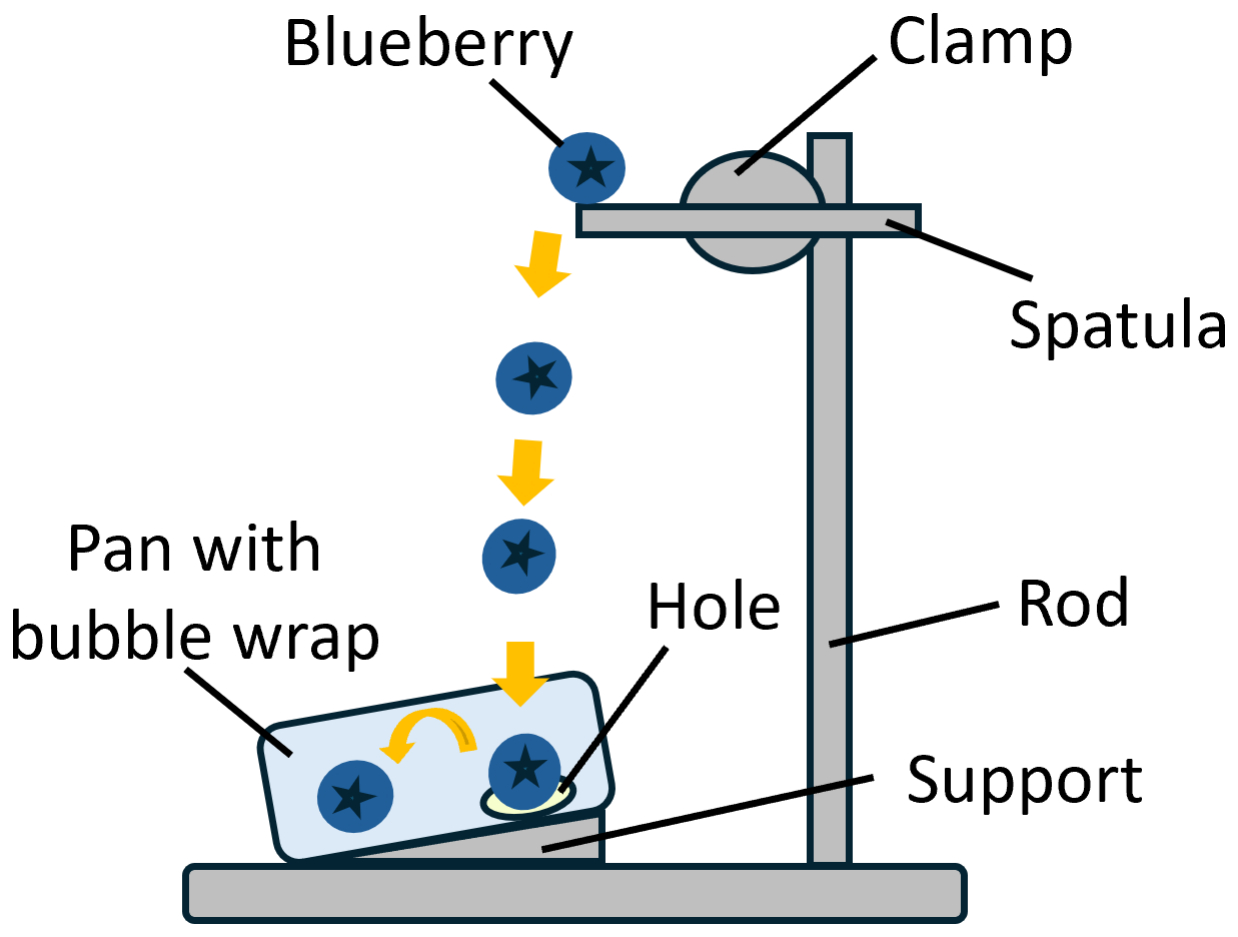
Illustration of the mechanism to manually induce berry bruises by dropping the fruit to a pan.

**Figure 3:**
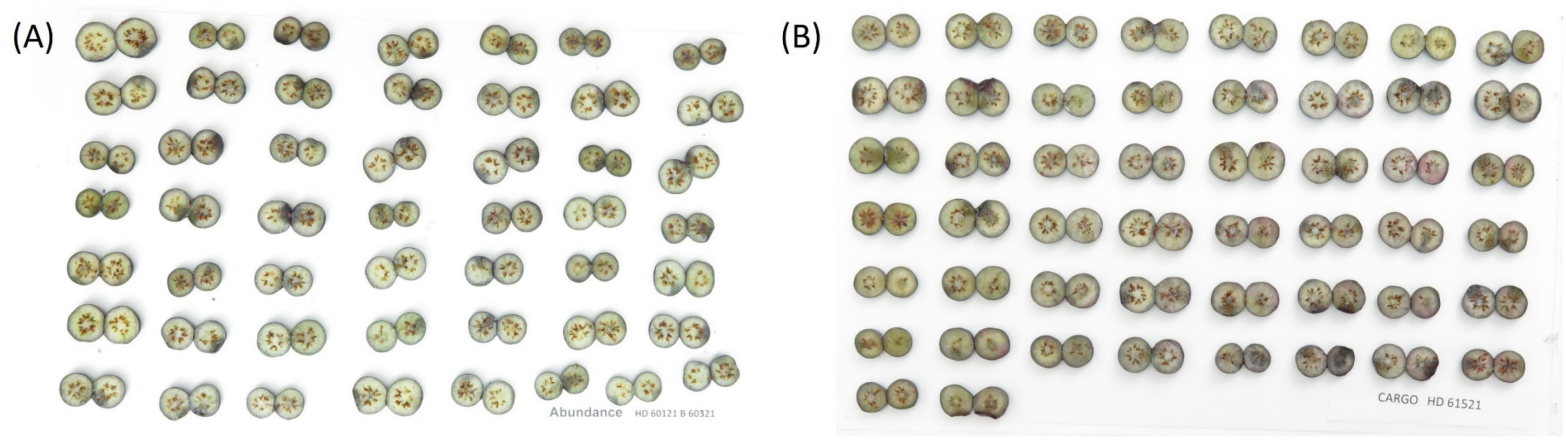
Example images of different cultivars. **(A)** Abundance **(B)** Cargo.

Blueberries were collected from the Castle Hayne Research Farm, NC in 2021, 2022, and 2023 and transported on re-freezable ice in coolers to Kannapolis NC. A total of 61 cultivars were collected over three years, with 46 cultivars having complete data for all three years. All blueberries were held at 4°C overnight then warmed to 15°C and sorted as firm and fully blue fruit of similar berry sizes within cultivars. To generate internal bruising for each blueberry, blueberries that were completely blue and firm were individually dropped 20 cm onto a 300 series stainless steel pan (6.62 L, 26x38x10.5 cm) (**Figure 2**). Blueberries were placed on the equatorial side and gently rolled off the end to drop onto the stainless-steel pan placed at a 15° angle to ensure the contact surface was around the equator of blueberries. The pan was lined with a layer of bubble wrap and a 9 cm diameter hole was cut out of the wrap to ensure that each blueberry hit only once on the pan and bounced to the bubble wrap. Fifty to sixty bruised berries of one cultivar were held for 18 hours at room temperature (22-23°C, 55% RH) in plastic bags. Blueberries were then cut in half between the stem and calyx end with single-sided razor blades, and the two halves per berry were placed on white paper. Each cultivar was photographed with a digital camera (**Figure 3**).

Another set of blueberries was separated into three groups, each consisting of 10 blueberry replicates, which were then placed in 163 ml plastic deli cups. Room-temperature blueberries were individually tested using a TA.XTPlus Texture Analyzer (Stable Micro Systems, Hamilton, MA, USA), which is equipped with a 2 mm flat probe (Oh et al., 2024a). A force-deformation profile was created for each fruit by penetrating the fruit on the equatorial axis using the following settings: a pre-test speed of 1 mm s^–1^, an auto-trigger force of 0.05 N, a test speed of 2 mm s^–1^, a stopping position at 90% strain, a post-test speed of 10 mm s^–1^, and a data collection rate of 200 points per second. The mechanical texture parameter YM20_BrSt (MPa/%) was calculated from the texture profile by determining the slope between the force at the minimum time and the force at 20% of burst strain (strain at maximum force).

### 2.3 Dataset establishment for model training

**Figure 4:**
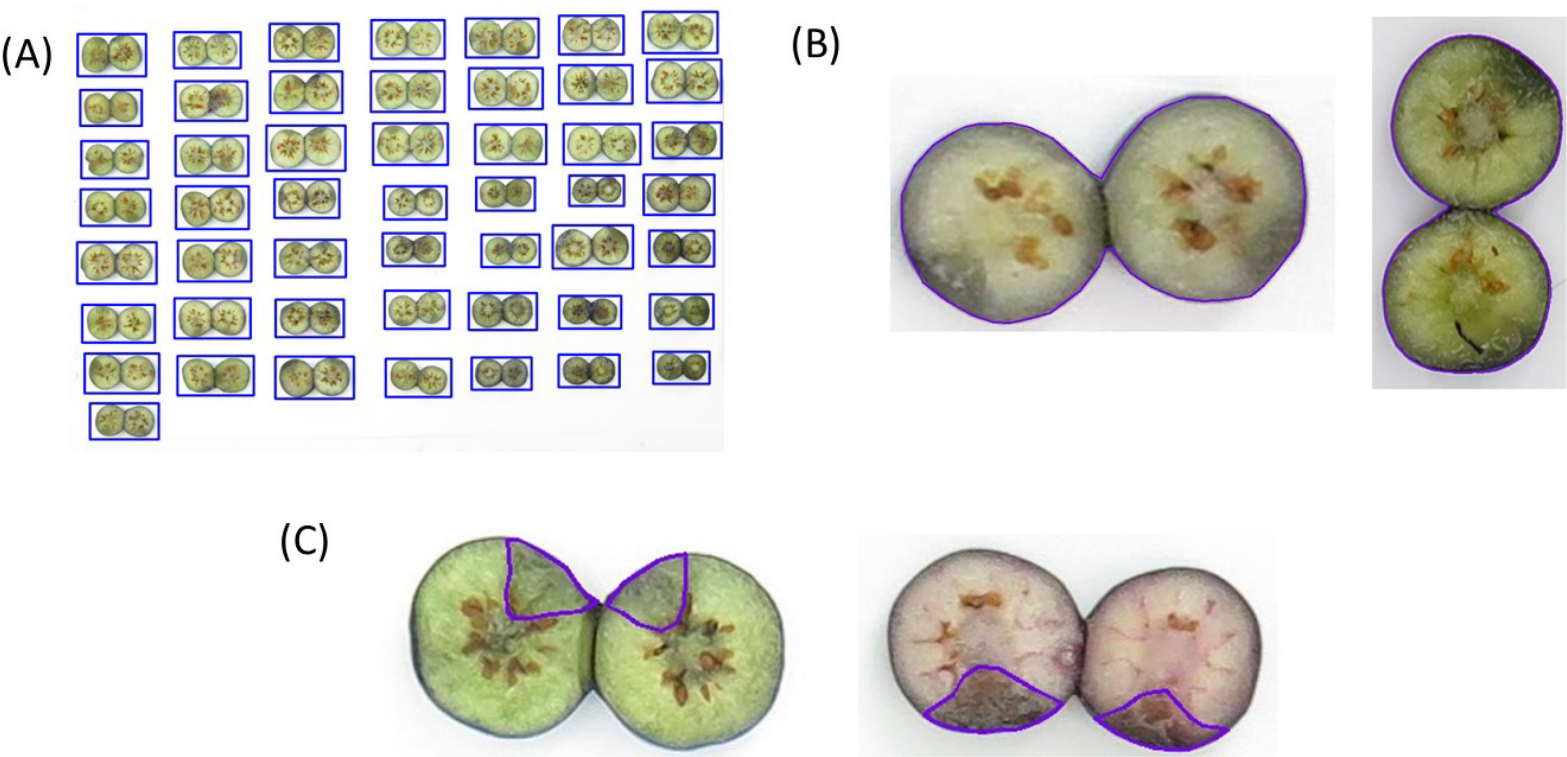
Annotated images for berry fruit detection **(A)**, fruit segmentation **(B)**, and bruised area segmentation **(C)**.

Three datasets were established for blueberry fruit detection, segmentation, and internal bruising detection to calculate blueberry internal bruising ratios. A total of 185 images of about 50 individual blueberries per image were annotated using RoboFlow (https://roboflow.com) with bounding boxes for individual berry detection (**Figure 4A**). Then all images were randomly split into training, validation, and testing with a ratio of 8:1:1. After that, the training images were augmented three times with flipping, rotation, and changing the saturation, brightness and exposure to enhance the diversity of the dataset, resulting in 447 training images. Images (162) of individual blueberries were randomly cropped from the annotations and annotated using RoboFlow with polygons for individual berry segmentation (**Figure 4B**). All images were used in the same ratio mentioned above to generate the dataset. Additionally, the training images were augmented three times with flipping and rotation, creating 478 images for training. The details of these two datasets are shown in **Table 1**.

**Table 1:** Datasets used for berry fruit detection and segmentation.

Dataset	Training	Validation	Testing
Fruit detection	447	18	18
Fruit segmentation	478	20	20

To build a dataset for internal bruising segmentation, two datasets were developed, with one using two years of images and the other using three years. The two-year dataset consisted of 676 individual berry images from 2021 and 2022. The three-year dataset included a total of 1001 images, comprising the same 676 images from 2021 and 2022 as the two-year dataset, along with an additional 325 images from 2023. All images were annotated using RoboFlow with the polygons to represent the machinal bruising area (**Figure 4C**). All images were randomly split into training, validation, and testing with a ratio of 8:1:1. Moreover, the training images of two datasets were also augmented three times with flipping and rotation, leading to 1614 and 2390 images for training, respectively. The details of these two datasets used for internal bruising segmentation are provided in **Table 2**.

**Table 2:** Datasets used for internal bruising segmentation.

Data source (year)	Training images	Validation images	Testing images
2021, 2022	540 (1614, aug^z^)	68	68
2021, 2022, 2023	799 (2390, aug^z^)	101	101

z aug represents augmentation and the number before represents the number of images after augmentation.

In addition, the bruising ratios of 101 testing images were calculated based on the annotated masks of blueberry and internal bruising as the ground truth, which were compared with the bruising ratio calculated based on the predicted masks of blueberry and internal bruising.

### 2.4 Models

**Figure 5:**
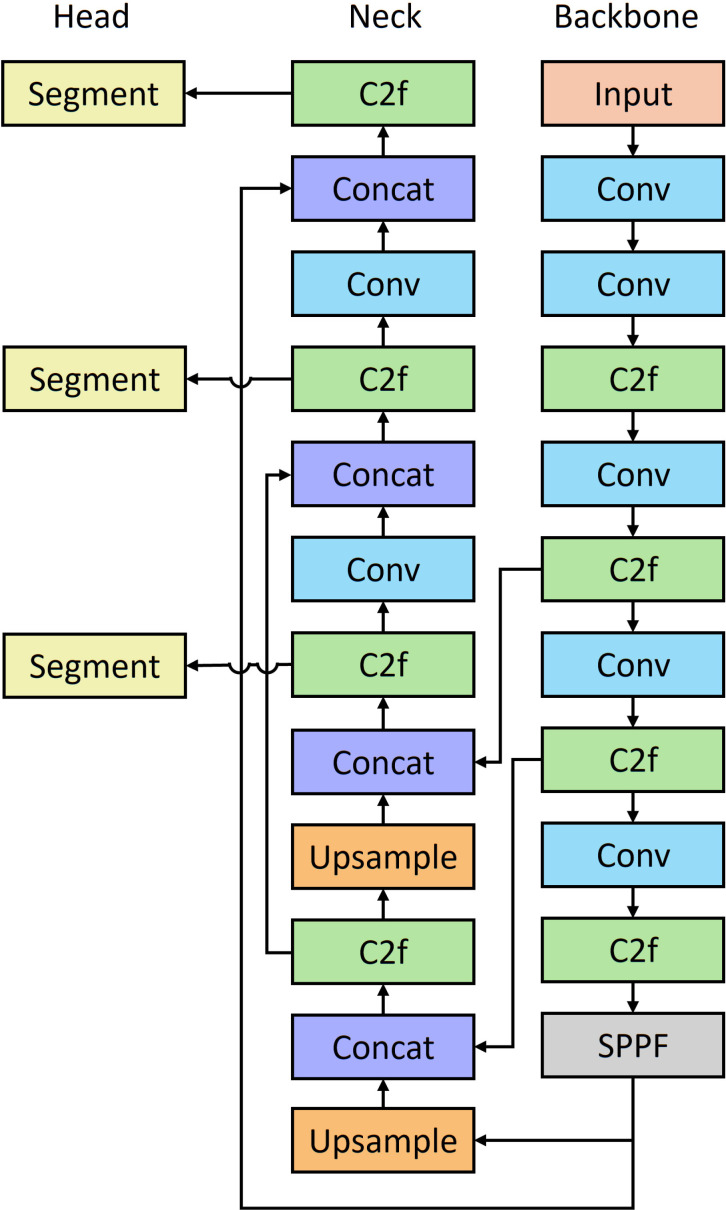
The architecture of YOLOv8 segmentation model.

The YOLO model is well known for its balance between speed and accuracy. It has been widely used for object detection and object segmentation in the agricultural domain. The YOLOv8 detection model was designed to detect objects with bounding boxes and the segmentation model was designed to segment the boundaries of each object, enabling more accurate measurements and analyses. In this study, we employed YOLOv8 detection model for individual blueberry detection, which can help locate the individual blueberry with bounding box. Subsequently, the segmentation model was applied to segment both the external boundary and internal bruising of each detected blueberry. The YOLOv8 for detection and segmentation (**Figure 5**) has a very similar architecture. The backbone and neck of these two models are the same. Both of them employed the C2f module to build the CSPDarknet backbone. Moreover, feature pyramid networks (FPN) and path aggregation networks (PAN) were leveraged in the neck to enhance the feature fusion over different scales. The difference between detection and segmentation is the head.

**Figure 6:**
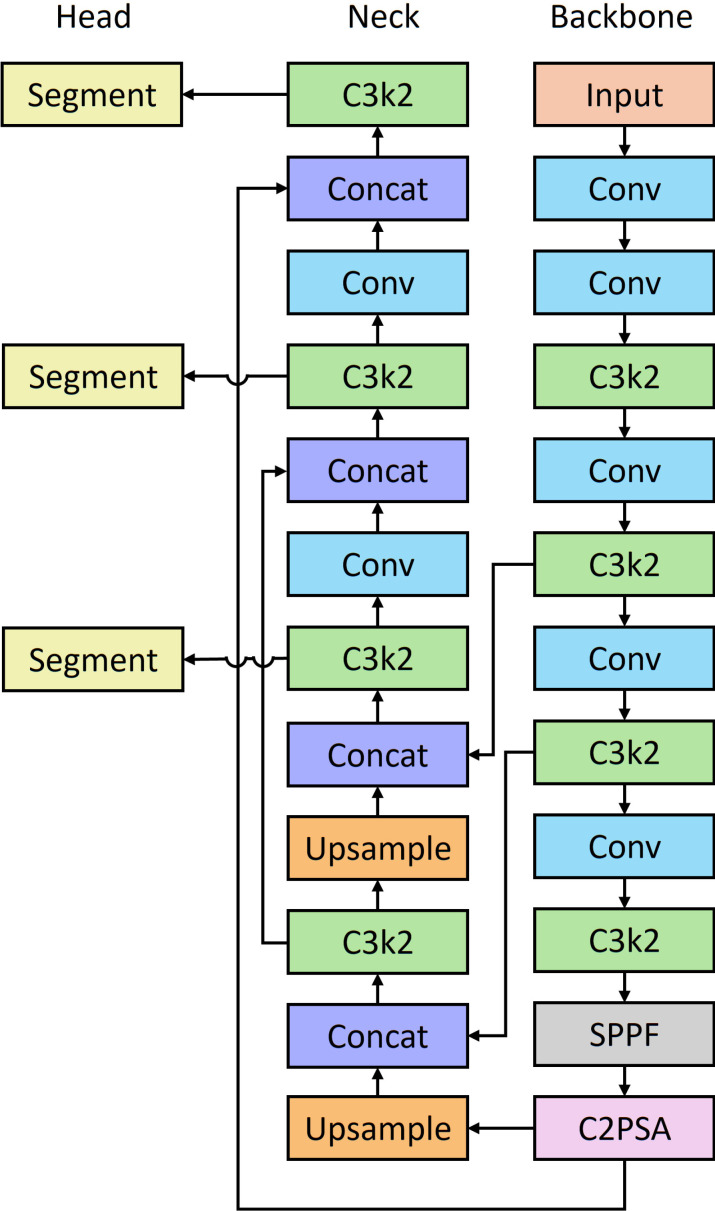
The architecture of YOLO11 segmentation model.

In the YOLOv8 detection model, two convolutional branches were employed to predict the bounding box and class, respectively. In the YOLOv8 segmentation model, these two branches were reserved, and an extra mask branch was added, which followed the principles of YOLACT (Zhao et al., 2023). The mask branch was leveraged to predict prototypes with mask coefficients. Mask coefficients and prototypes were combined using a linear combination of the latter with the former as coefficients to produce masks (Bolya et al., 2019). YOLO11 was used for internal bruising segmentation because mechanical bruising is more challenging compared to blueberry segmentation. C2f was replaced by C3k2 in YOLO11 (**Figure 6**) to maintain efficient feature extraction while providing more flexible configurations to adapt to different computing needs. Additionally, a C2PSA layer was added to enhance spatial attention in the feature maps, which allows the model to focus on relevant image regions. These improvements increased the performance of YOLO11. As the tasks of individual blueberry detection and segmentation were relatively simple and YOLOv8 performed well on these two tasks, YOLO11 was not employed for these two tasks.

After obtaining the masks of individual berries and internal bruising, the bruising ratio was calculated by dividing the pixel number of the berry region by the pixel number of the bruising region. The mean bruising ratio of one cultivar was then calculated by averaging the bruising ratios of all samples. To distinguish the bruise-resistant and bruise-susceptible cultivars, the K-means clustering algorithm was applied to the mean bruising ratio of each cultivar for each year, with K set to 2. To validate the separation, Welch’s t-test was conducted to test the null hypothesis that there was no significant difference between the two clusters. Welch’s t-test was chosen for its robustness against unequal variances and sample sizes, ensuring a more reliable comparison between bruise-resistant and bruise-susceptible cultivars.

### 2.5 User interface design

**Figure 7:**
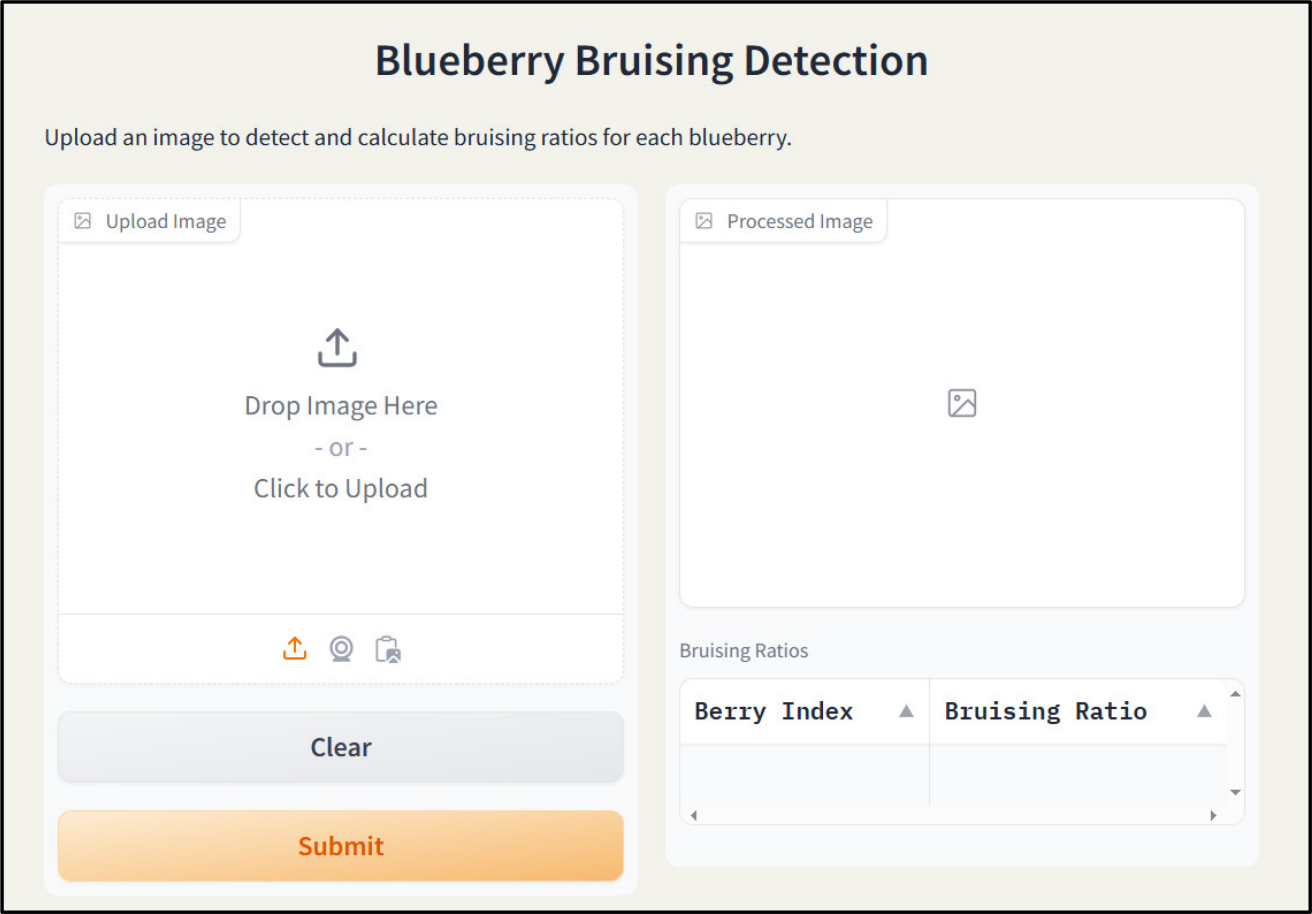
Snapshot of the blueberry bruising detection web app.

To combine three models and make them easier to use, a user-friendly interface was developed based on Gradio. Gradio is a Python library that can be used to create web-based interfaces for machine learning models. It supports inputs, such as text, image, and video, and outputs such as image, text, and table. Additionally, it also provides a shareable link to the created application for quick testing by users. Moreover, it also can integrate with Hugging Face for hosting models and apps. The developed blueberry internal bruising detection user interface contains two main functional modules (**Figure 7**). The left part is used to upload images for processing. After selecting an image and clicking the “Submit” button, the program automatically allocates the trained models to detect and segment individual fruits and then segment the internal bruising. By obtaining the mask of the individual berry and its internal bruising, the bruising ratio is calculated by dividing the pixel number of the individual berry by the pixel number of bruising. The processed image returns to the interface and shows in the right “Processed Image” module. Additionally, the berry index with bruising ratio returns to the right table below as well.

### 2.6 Training configurations

All models were trained on the HiperGator high-performance computing cluster, which included 8 AMD EPYC ROME CPU cores, 1 NVIDIA DGX A100 GPU (80 GB), and 32 GB of memory. The operating system is Linux, with essential software libraries including Python 3.10, PyTorch 2.4.1, ultralytics 8.3.3, CUDA 12.1, OpenCV 4.10, and Gradio 4.44.1.

The pre-trained weights on the COCO dataset were employed when training the individual blueberry detection and segmentation models, which can benefit the training of a small dataset by leveraging the knowledge learned from a large dataset to improve the model performance and reduce training epochs. To train the internal bruising segmentation models, YOLOv8 and YOLO11 segmentation models were trained on 2-year data and 3-year data, respectively. Additionally, to evaluate the effectiveness of pre-training and data augmentation, each model was trained with and without pre-training and augmentation, respectively.

### 2.7 Evaluation metrics

To evaluate the detection and segmentation performance, P (Precision), R (Recall), and mAP were employed in this paper. Precision measures the proportion of true positive detections among all detections. Recall measures the proportion of true positive detections relative to the total ground truth detections. Average Precision (AP) captures the overall shape of the precision/recall curve, evaluating both precision and recall comprehensively. The mAP is calculated by averaging the AP values across all classification categories. Moreover, linear regression tests were employed in this study to evaluate the correlation between the predicted bruising ratio and the ground truth, as well as the correlation between the bruising ratio and YM20_BrSt with evaluation metrics including the fitted slope, coefficient of determination (R^2^), and Root Mean Squared Error (RMSE). Furthermore, MAPE was also calculated to evaluate the performance between the predicted bruising ratio and ground truth. The calculations of the metrics are shown in Equations 1–6.


(1) $$P=\frac{TP}{TP+FP}$$

(2) $$R=\frac{TP}{TP+FN}$$

(3) $$AP=\int_{0}^{1}P\left(R_{i}\right)dR_{i}$$

(4) $$mAP=\frac{1}{C}\sum_{n=1}^{C}AP_{i}$$

(5) $$RMSE=\sqrt{\frac{\sum_{0}^{N}{\left(Pf_{i}-P_{i}^{\prime }\right)}^{2}}{N}}$$

(6) $$MAPE=\frac{1}{N}\sum_{0}^{N}\left|\frac{P_{i}-P_{i}^{\prime }}{P_{i}}\right|$$

where, *TP*, *FP*, and *FN* represent true positives, false positives, and false negatives, respectively. *C* denotes the number of class categories. $AP_{i}$ represents the area under the P-R curve of the *i*th class. N denotes the total number of samples. $Pf_{i}$ , $P_{i}^{'}$ and $P_{i}$ represent the predicted result from fitted curve, predicted bruising ratio and ground truth of the *i*th sample, respectively.

## 3 Results

### 3.1 Results of blueberry fruit detection and segmentation

**Figure 8:**
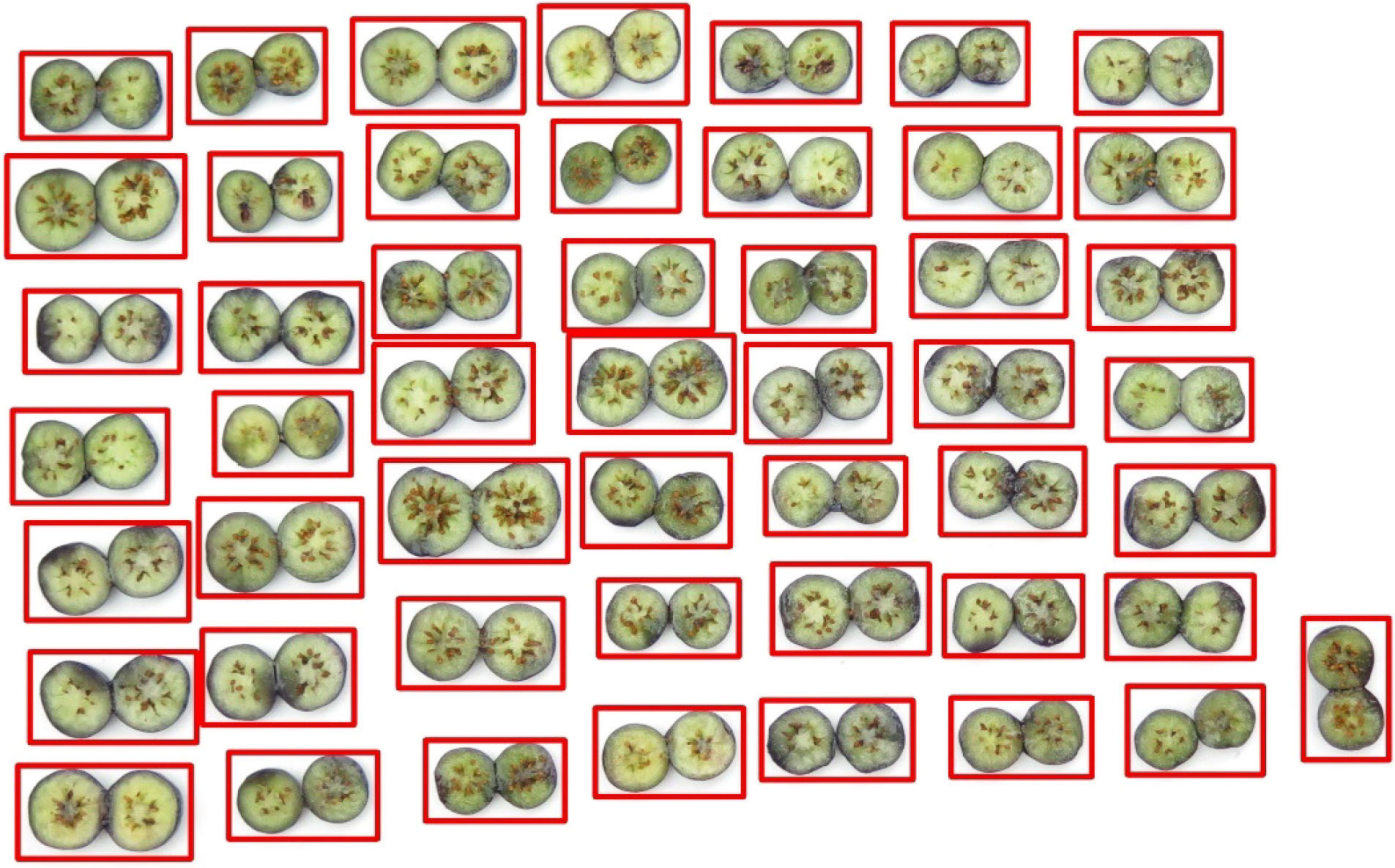
Blueberry fruit detection result.

**Figure 9:**
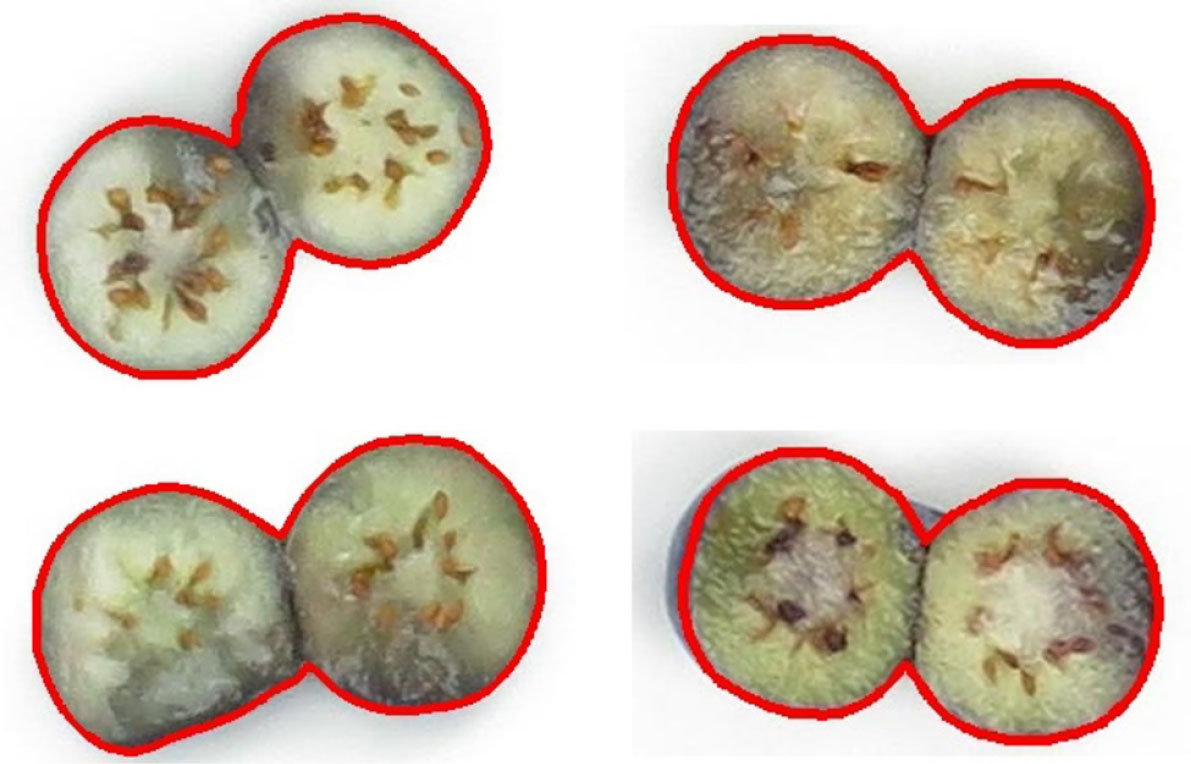
Blueberry fruit segmentation result.

The performances of individual blueberry detection and segmentation were evaluated on the testing datasets and the results illustrate that the trained models achieved very high accuracy (**Table 3**). Specifically, the blueberry detection achieved an mAP0.5 of 0.995 and 0.993 on a stricter metric mAP0.5:0.95. The qualitative result demonstrated in **Figure 8** also showed that the trained blueberry detector can detect each blueberry with a bounding box correctly, even the vertically placed sample. The segmentation model also achieved a high mAP0.5 of 0.995 and an mAP0.5:0.95 of 0.990. Although bruising or compression by dropping the fruit on a hard surface may cause deformation and create the irregular shape of the blueberry fruit, the segmentation model still can obtain the correct mask of the cross-section area (**Figure 9**).

**Table 3:** Performance on blueberry fruit detection and segmentation.

Task	P	R	mAP0.5	mAP0.5:0.95
Blueberry detection	1	1	0.995	0.993
Blueberry segmentation	1	1	0.995	0.990

### 3.2 Results of bruised tissue segmentation

**Figure 10:**
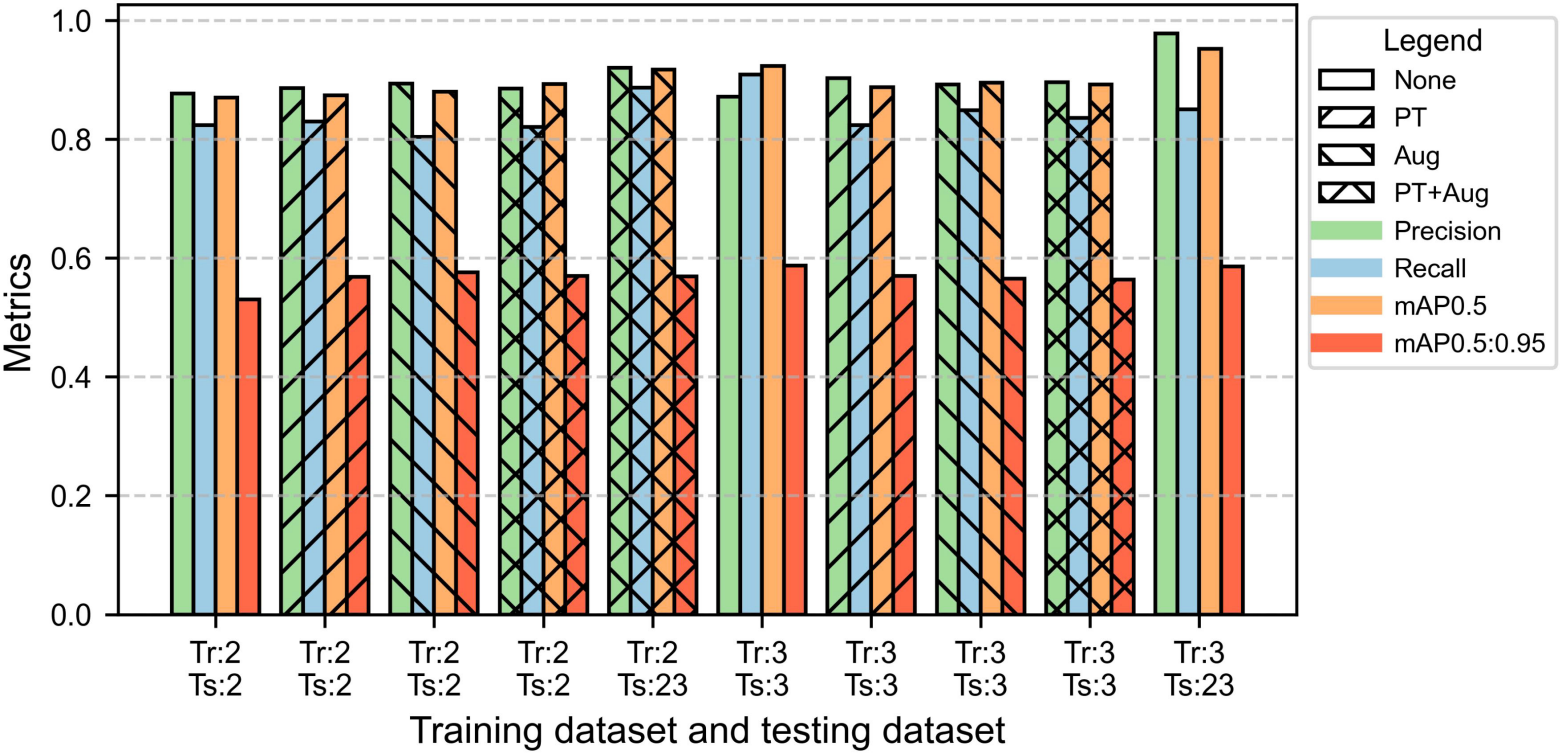
Performance of bruising segmentation using YOLO8-seg. Tr and Ts represent training dataset and testing dataset, respectively. The numbers 2 and 3 represent the datasets from 2-year data and 3-year data, respectively, and 23 represents the dataset from the year 2023. None represents without using both pre-trained weights and augmented training dataset. PT represents using pre-trained weights from the COCO dataset when training. Aug represents using the augmented training dataset for training. All the tests were performed on the corresponding testing datasets.

**Figure 11:**
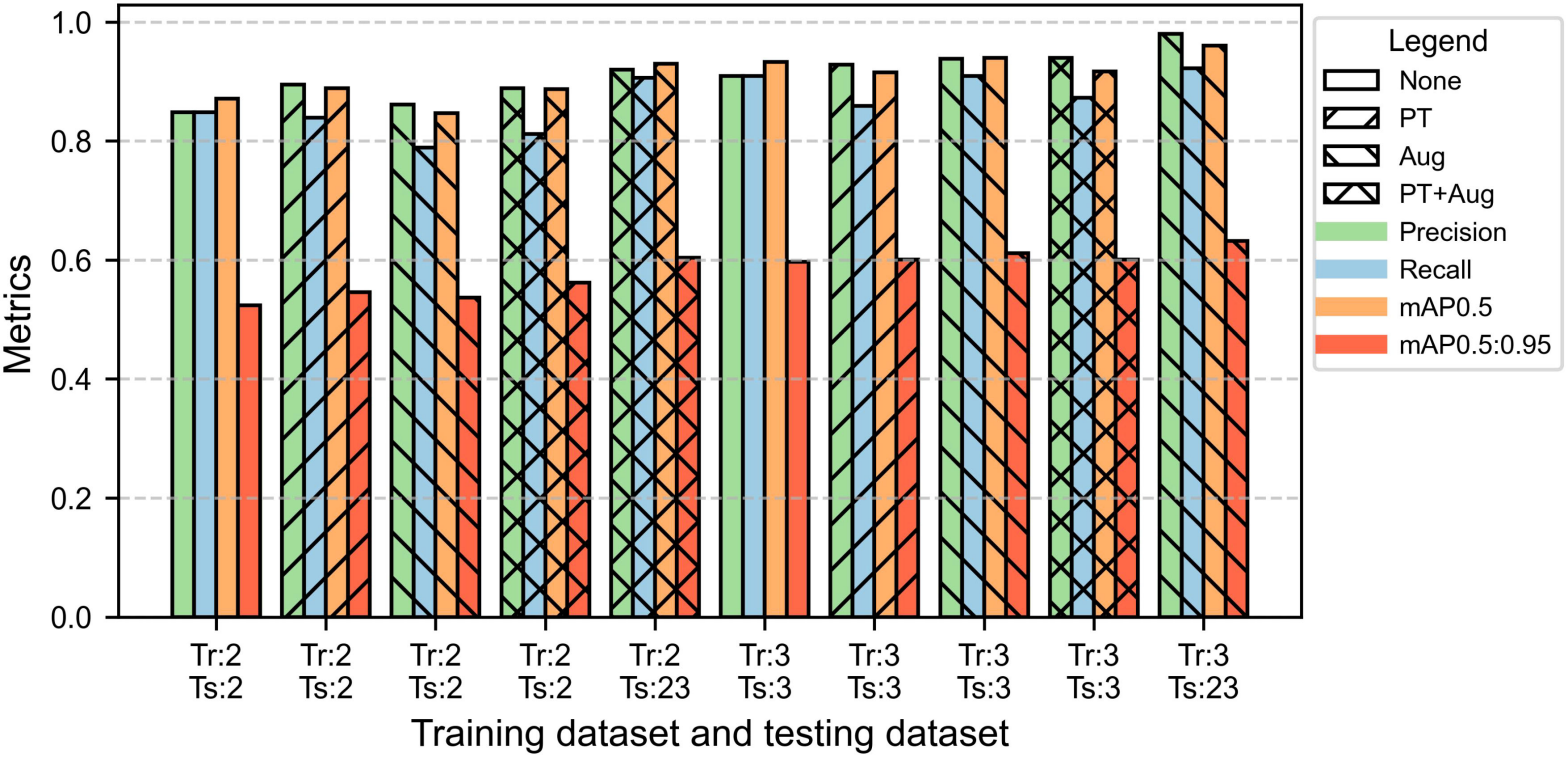
Performance of bruising segmentation using YOLO11-seg. Tr and Ts represent training dataset and testing dataset, respectively. The numbers 2 and 3 represent the datasets from 2-year data and 3-year data, respectively, and 23 represents the dataset from the year 2023. None represents without using both pre-trained weights and augmented training dataset. PT represents using pre-trained weights from the COCO dataset when training. Aug represents using the augmented training dataset for training. All the tests were performed on the corresponding testing datasets.

Comparisons between two datasets (2-year data and 3-year data) and two models (YOLOv8 and YOLO11) revealed that using 3-year data significantly improved the segmentation performance of YOLO models for internal bruising, with YOLO11 outperforming YOLOv8 (**Figures 10**, **11**). The results showed that both YOLOv8 and YOLO11 achieved higher performances when they were trained on 3-year data. Compared to the best model trained on 2-year data, the best models of YOLOv8 and YOLO11 trained on 3-year data increased 3% and 5.1% on mAP0.5, respectively. The best model of YOLO11 achieved an mAP0.5 of 0.940 on 3-year data, which was 4.7% higher than that of YOLOv8.

**Figure 12:**
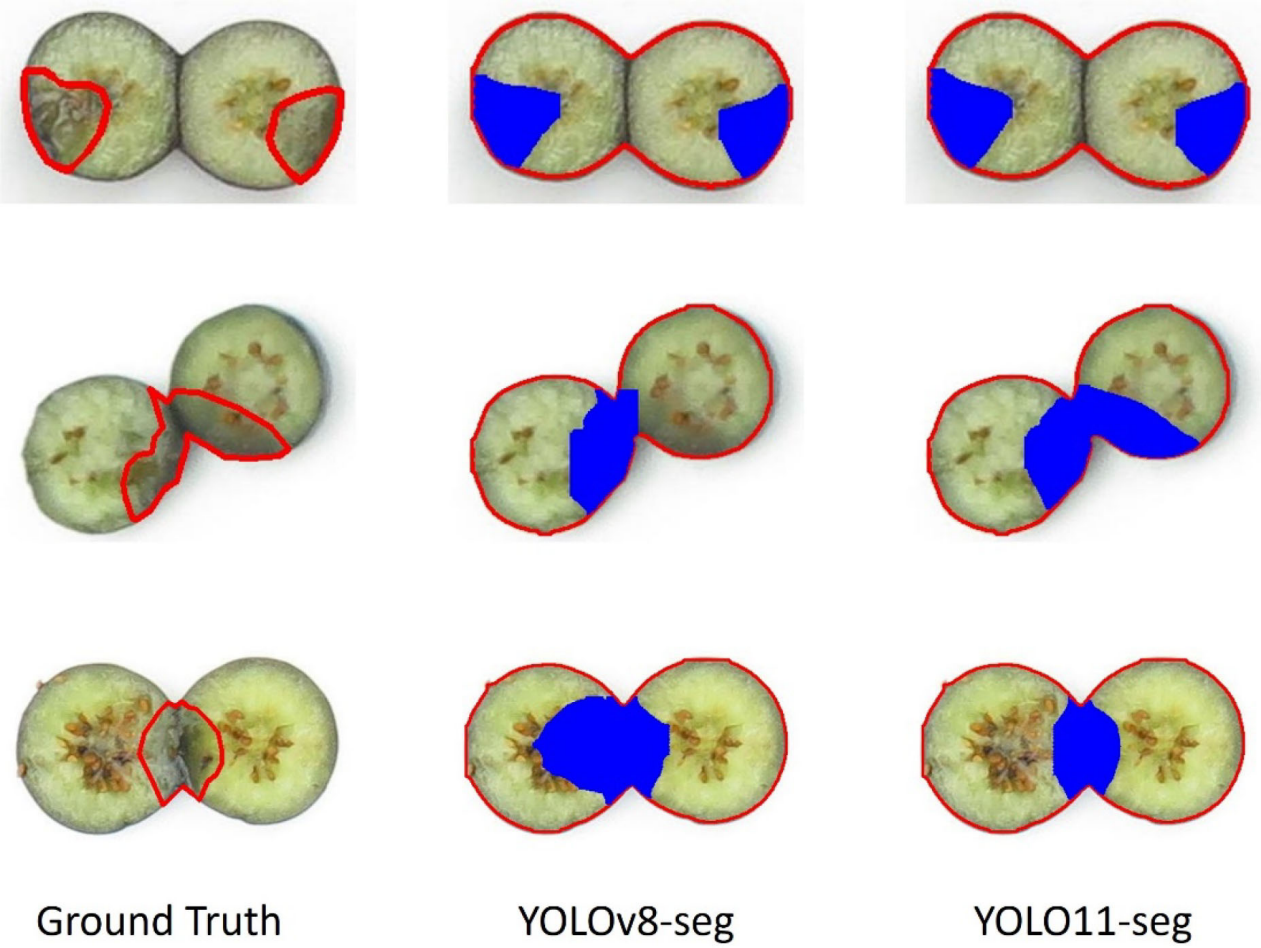
Examples of bruised area segmentation results.

The comparison between YOLOv8 and YOLO11 indicated that the polygons of the bruised tissue predicted by YOLO11 were closer to ground truth compared to those predicted by YOLOv8 (**Figure 12**). Additionally, the segmentation model trained on 2-year data also exhibited the generalization ability to segment bruising on the data from 2023. The YOLOv8 and YOLO11 models trained on 2-year data achieved mAP0.5 of 0.917 and 0.930 on the 2023’s testing dataset, which were 3.5% and 3% lower than those models trained on 3-year data, respectively. Additionally, the effectiveness of pre-training and augmentation techniques were also evaluated when training the model. In **Figure 10**, pre-training and augmentation improved the mAP0.5 on 2-year data by 2.3% compared to the model without using these two techniques. A similar trend was also observed in YOLO11; mAP0.5 increased from 0.871 to 0.887, although using the augmentation may cause the mAP0.5 to slightly decrease. Using a larger dataset containing 3 years’ data, pertaining and augmentation techniques did not improve the segmentation performance of YOLOv8. However, when only using augmented 3-year data to train YOLO11, the mAP0.5 slightly increased (1%) compared to the model without using augmentation. Overall, using all 3-year data achieved better segmentation performance. Additionally, YOLO11 outperformed YOLOv8 for internal bruising segmentation. Moreover, the pre-training and augmentation may be more effective on 2-year data compared to 3-year data.

### 3.3 Results of bruising ratio and analysis across three years

**Figure 13:**
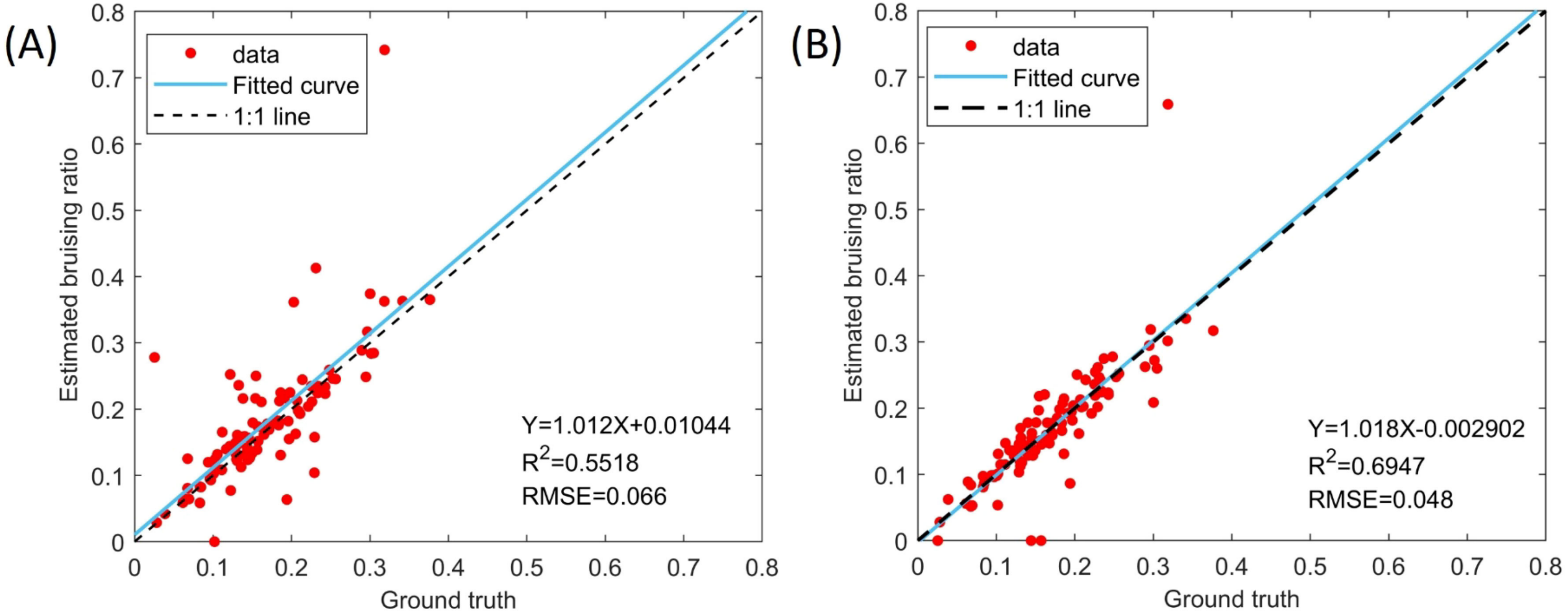
Comparison of predicted bruising ratio with the ground truth. **(A)** YOLOv8-seg and **(B)** YOLO11-seg.

**Figure 14:**
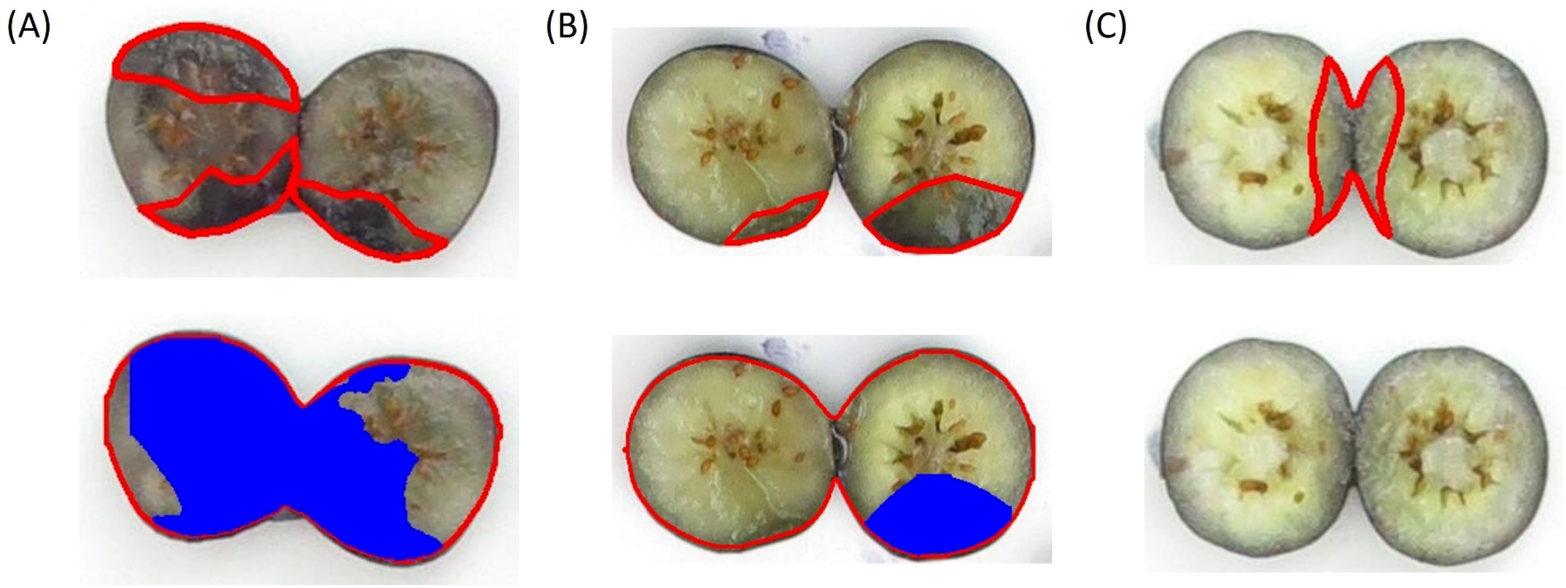
Incorrect segmentation examples. The first row is the ground truth and the second row is the segmentation results. **(A)** represents false positives and **(B, C)** represent false negatives.

The best-performing models of YOLOv8 and YOLO11 trained on 3-year data were leveraged to process 101 testing images and the comparison between the predicted bruising ratios and ground truth demonstrated that YOLO11 achieved higher accuracy than YOLOv8 in predicting bruising ratios. The linear regression results showed that the calculated bruising ratios of YOLO11 were closer to the ground truth compared to those of YOLOv8 (**Figure 13**). Specifically, the R^2^ of YOLO11 was 0.14 higher than that of YOLOv8. The RMSE of YOLO11 was about 0.02 lower than that of YOLOv8. In addition, the MAE and MAPE of YOLO11 were 0.025 and 15.87%, which were 0.009 and 12.29% lower than those of YOLOv8. However, a sample containing a discolored area caused by impact and other factors (**Figure 14A**) led to overestimations by both models, resulting in significant errors in the comparison results. Overall, the bruising ratios calculated based on the predictions of YOLO11 outperformed YOLOv8, which was consistent with the evaluation results of bruising segmentation.

**Figure 15:**
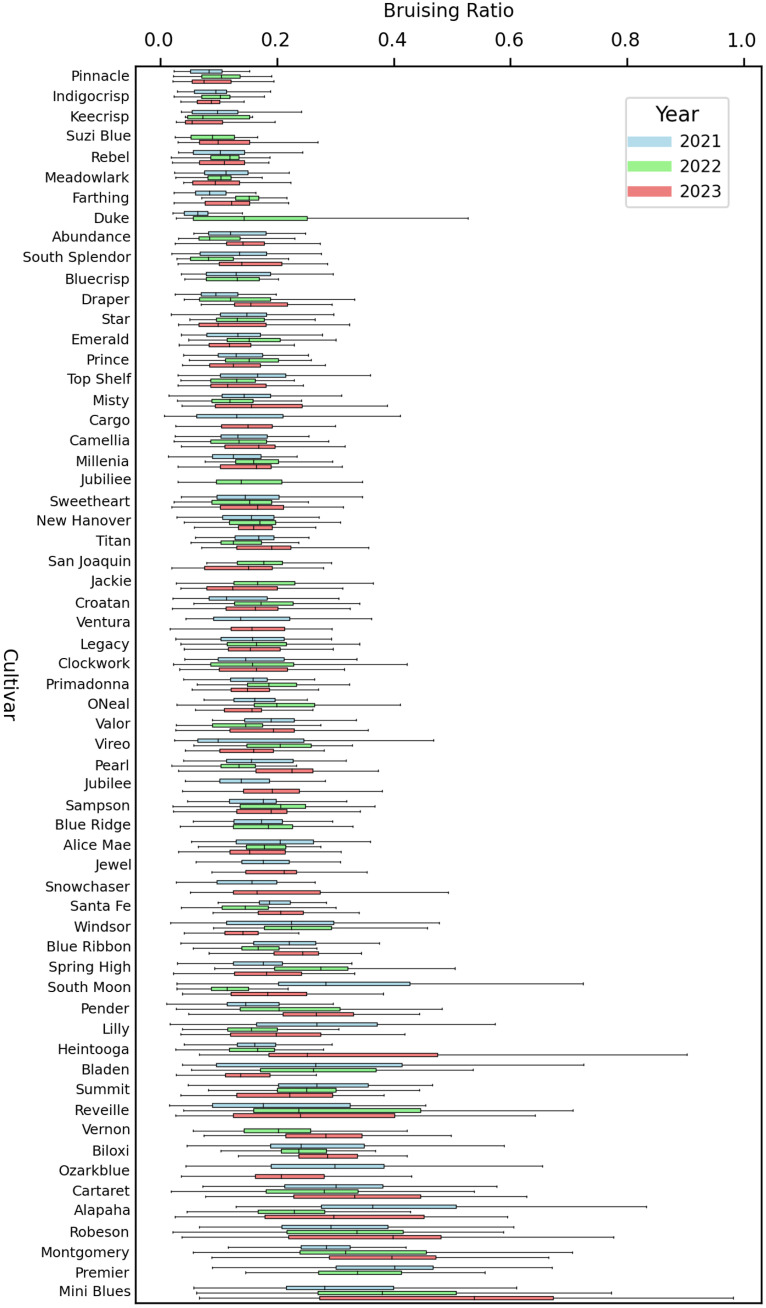
A comparison of bruising ratio distribution of 61 blueberry cultivars, and 46 cultivars have all three-year data. Error bars extend to the minimum and maximum values, excluding outliers. Boxes indicate the interquartile range, which contains the middle 50% of the data points for each genotype in a given year. The center line inside each box indicates the median bruising ratio for that year.

Analysis of blueberry bruising across cultivars and years using the YOLO11 segmentation model revealed significant variability in bruise susceptibility, with some cultivars consistently demonstrating low bruising ratios while others exhibited high bruising tendencies and considerable year-to-year variation (**Figure 15**). For example, cultivars like “Indigocrisp” and “Pinnacle” (considered crisp and firm cultivars) maintain relatively low bruising ratios over years, making them potential candidates for breeding programs targeting for enhancing suitability for machine harvesting. In contrast, cultivars such as “Robeson”, “Montgomery”, and “Mini Blues” (well known to be soft cultivars) maintained relatively high bruising ratios over years, reflecting their greater susceptibility to bruising. Additionally, many cultivars showed clear year-to-year variation. For instance, “Heintooga” demonstrated a pronounced increase in bruising ratio in 2023. Similarly, “South Moon” also showed inconsistent performance across the years. On the other hand, “Pinnacle” and “Indigocrisp” exhibit relatively low bruising ratios over time, suggesting a higher level of resilience to external factors such as environmental conditions or handling practices during harvest. Within-year variation further distinguishes the cultivars. For instance, some cultivars, such as “Rebel” and “Keecrisp”, show tight distributions within a single year, indicating consistent bruising ratios among samples. Conversely, cultivars such as “Premier” and “Mini Blues” exhibit wide variation within a year, suggesting varied responses to environmental or postharvest handling conditions. Although blueberry fruits were carefully selected for full color and firmness, blueberries exhibit asynchronous ripening. It is possible that some fruits were slightly riper than others within the same harvest, or that softening is slightly more accelerated for some cultivars (especially the softer types) than others.

**Figure 16:**
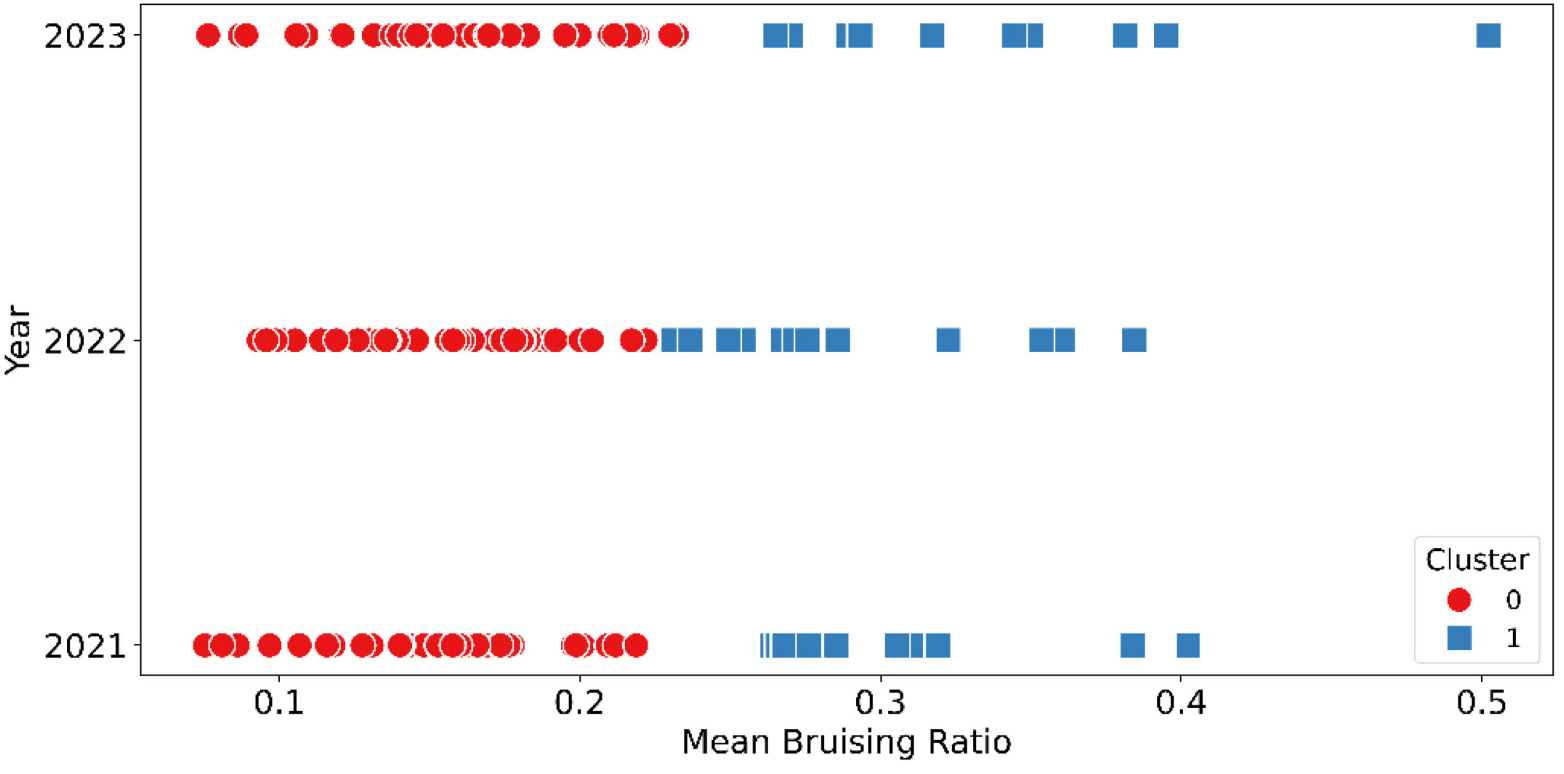
K-means clustering results of mean bruising ratio over three different years.

Moreover, the K-means clustering algorithm was employed on mean bruising ratios to distinguish bruise-resistant and bruise-susceptible cultivars and the results showed that a threshold of 0.22 could be used to separate these two groups of cultivars (**Figure 16**). The clustering result of 2021 showed that the distinction between clusters was clear, with Cluster 1 containing many genotypes exceeding a mean bruising ratio of 0.25, while Cluster 0 clusters below 0.20. The clustering result of 2023 retained a similar distribution to 2021 although the gap between two clusters narrowed. The gap between clusters further narrowed among 2022’s data and the distinction between the clusters becomes less pronounced compared to 2021 and 2023. The t-test results, with extremely low p-values (2021: 1.33e-08, 2022: 2.23e-07, and 2023: 1.65e-5), confirm significant differences between the two clusters identified by K-means across all three years.

**Figure 17:**
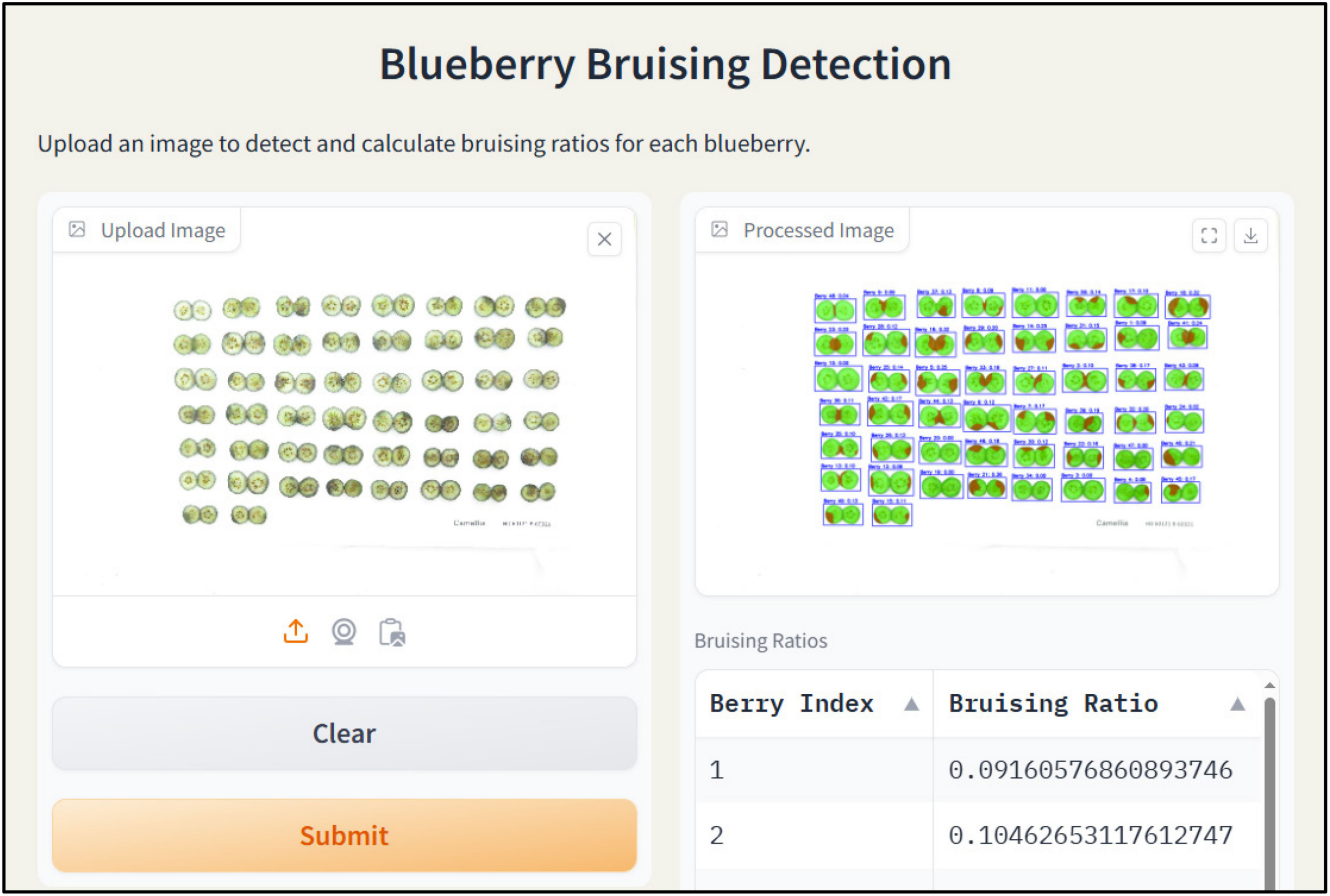
The graphical user interface (GUI) of the blueberry bruising detection web app with a raw image (left panel) and a processed image (right panel).

A snapshot of the developed web app with a processed image was illustrated in **Figure 17**. The left shows the uploaded image for processing. The processed image returns to the interface on the right with the bounding box that separates each blueberry with the index and bruising ratio shown above the bounding box. Additionally, the green mask and red mask shown on each blueberry represent the healthy region and the bruising region, respectively. The index with bruising ratio also returns to the table below, facilitating easier saving and further analysis.

### 3.4 Results of the correlation between bruising ratio and YM20_BrSt

**Figure 18:**
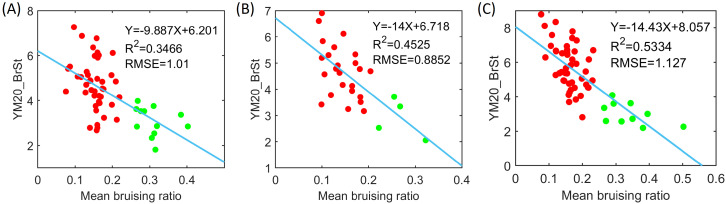
Correlation between bruising ratio and Young’s modulus parameter, YM20_BrSt, over three years: **(A)** 2021, **(B)** 2022, and **(C)** 2023. Red dots represent bruise-resistant cultivars and green dots represent bruise-susceptible cultivars that were categorized by clustering results.

The linear regression tests between the calculated mean bruising ratio and YM20_BrSt exhibited a moderate correlation and the mean bruising ratio is inversely proportional to YM20_BrSt (**Figure 18**). Specifically, a moderate correlation (R^2^ = 0.35) with an RMSE of 1.01 was observed in 2021’s data. 2022’s data displayed a stronger correlation (R^2^ = 0.45) with the lowest RMSE (0.89). 2023’s data exhibited the strongest correlation (R^2^ = 0.53) but had a slightly higher RMSE (1.1). Overall, the relationship between the mean bruising ratio and YM20_BrSt was consistent over three years and the observed variations may relate to the sample characteristics or experimental conditions. Additionally, we mapped the clustering results based on bruising ratio to the YM20_BrSt values. Cultivars with higher YM20_BrSt values exhibit greater resistance to bruising, while those with lower YM20_BrSt values are more susceptible. Furthermore, Welch’s t-test was performed on the YM20_BrSt values of the two categories identified through clustering. The t-test results showed low p-values (2021: 3.54e-07, 2022: 0.008, and 2023: 1.06e-10), indicating significant differences between the two categories of YM20_BrST values identified through the mean bruising ratios.

## 4 Discussion

This paper demonstrated a deep learning-based high-throughput approach for blueberry internal bruising detection on RGB images, which can benefit the breeding of bruise-resistant cultivars for mechanical harvesting. Additionally, a user-friendly interface was designed to easily use the trained models. A batch of blueberries can be analyzed simultaneously, which increases the processing efficiency. Moreover, the developed approach does not require expensive imaging systems, such as hyperspectral cameras and special lighting conditions, and is suitable for fast and portable detection with only RGB cameras.

Although the developed approach achieved a high mAP0.5 in bruising segmentation and promising bruising ratio estimation results, the moderate correlation suggests room for improvement in predicting masks. A prediction is deemed a true positive if its overlap with the ground truth exceeds 0.5; however, this still allows for up to 50% of the prediction to be incorrect, potentially introducing errors in the estimated bruising area. Moreover, the R² value is particularly sensitive to samples with large errors, which further exacerbates the lower correlation. In addition, the developed approach still has a few potential limitations. For example, the bruising was generated by dropping the blueberry onto the pan. The bruising area was controlled to the equator of the fruit. To avoid the second bruising, the bubble wraps were used to catch the bounced fruit. However, the contact surface of the fruit was not always on the equator due to the spinning of the fruit during the dropping, which may cause an inaccurate bruising ratio prediction. Therefore, the bruising generation system could be further improved by restricting the orientation of the fruit during dropping to make sure the contact surface is along the equator. This approach also requires manual slicing of the blueberry along the equator to get two halves using the razor blade. If the slicing position does not align with the center of the bruised area, the bruising ratio may be underestimated, limiting the feasibility of this method for real-time field applications. A more convenient tool with fixture and automatic cutter could be designed and built to streamline the slicing process and enhance efficiency.

The performance of individual blueberry detection and segmentation was exceptionally high, even with a relatively small dataset. This could be attributed to the simplicity of the background, which lacked significant interference, despite variations in the shape, placement angles, and color of the blueberries. As a result, a small dataset was sufficient to achieve highly accurate results. However, if the background includes noise, the detection accuracy could be affected. Future work could include training and evaluating the model on images with varied and complex backgrounds to improve its robustness under diverse conditions. Additionally, the discolored area on the blueberry may not be generated by impact, which may affect the segmentation accuracy of the trained bruising segmentation model. The bruise caused by impact usually occurs on the impact point and then spreads to the inner tissue and the shape is like a triangle (**Figure 4C**). **Figure 14A** shows an example that contains both impact bruising and other bruising. Although there is a discolored area inside the core, this area is not caused by the impact bruising; the segmentation model still segmented this area as impact bruising, resulting in overestimation. Conversely, some small discolored areas (**Figure 14B**) and lightly discolored areas (**Figure 14C**) may be missed by the segmentation model, leading to underestimation of bruising. Moreover, variability in bruising susceptibility (the onset of the discolored area as the bruising symptom) due to environmental factors, such as temperature, humidity, and handling practices, may also impact the robustness of the model. More samples with complex bruising areas could be added to the training dataset in the future to further improve the segmentation accuracy. Compared to our previous research (Ni et al., 2022), the R² achieved in their study is 0.729, which is slightly higher. Our approach achieves a bruise ratio accuracy of 87.7%, surpassing their 79.0% due to the use of the latest segmentation model.

In this paper, only mature blueberries were examined in this study because only mature fruit was harvested, which is a typical practice in blueberry harvesting. The models’ performances that were trained with two-year data and three-year data were compared. Using only two-year data (2021 and 2022) to train the bruising segmentation model still achieved a high mAP on the 2023 data, which means the trained model had a generalization ability to segment bruising on new data. After using all three years of data, the results were further improved, which may be attributed to the enhanced data variability from images of 2023. Additionally, the pre-training and augmentation may be more effective on 2-year data compared to 3-year data. This is because the number of images in the 2-year dataset is relatively smaller than that in the 3-year dataset. Moreover, variability was observed between each cultivar over three years, which may have been caused by many factors. For example, different harvesting times over three years can result in slight maturity differences among blueberry samples within cultivars. Overripe (softer) blueberries are more likely to be bruised leading to a larger bruising ratio. In addition to maturity, the fruit size may also affect the bruising ratio. Earlier papers indicate that smaller berries exhibit more bruising (Yu et al., 2014), possibly because there is less volume to spread out the force that occurs upon impact. Moreover, factors such as berry morphology (relative roundness, peel thickness) and composition (cell wall thickness and components) may also affect bruise response.

Recent work demonstrated that the mechanical parameter YM20_BrSt is related to the firmness of blueberries (Mengist et al., 2024; Oh et al., 2024a; Oh et al., 2024b). In this study, we found a negative correlation between bruising ratio and YM20_BrSt, which supported a widely accepted assumption that firmer berries exhibit greater resistance to internal bruising. This highlights the potential of using instrumental measurements from a texture analyzer as indicators of bruise resistance that breeding programs could use for selection, particularly given the moderate to high heritability of mechanical texture in blueberry (Ferrão et al., 2024; Oh et al., 2025). However, it is important to note that the bruising ratio and measured firmness are not equivalent. Firmness reflects the mechanical resistance of fruit tissue to deformation while the bruising ratio quantifies the visible damage that occurs after impact. While firmer fruit generally tends to bruise less, the bruising ratio is influenced by additional factors such as tissue structure and internal composition. As shown in **Figure 18**, cultivars with similar YM20_BrST values (e.g., between 2 and 4) can display a wide range of bruising ratios, indicating that additional traits contribute to bruise susceptibility beyond firmness alone.

## 5 Conclusions

This study developed a high throughput blueberry bruising detection approach using deep learning models for sliced fruit. YOLOv8 models were used for individual blueberry detection and segmentation, which achieved high mAP for both detection and segmentation tasks. Additionally, YOLOv8 and YOLO11 segmentation models were compared for bruising segmentation and YOLO11 achieved higher segmentation accuracy by training on all three-year data with data augmentation. After analyzing more than 40 cultivars repeatedly over three years, the mean bruising ratio of 0.22 could be used as a target threshold for distinguishing bruise-resistant and bruise-susceptible cultivars. Moreover, the mean bruising ratio was negatively correlated with the mechanical firmness parameter YM20_BrSt, indicating that a texture analyzer could be useful in screening blueberry germplasm for bruise resistance. The developed user interface makes it easier for blueberry breeders, growers, and shippers/packers to access the deep-learning models. Overall, this study introduced an effective and efficient method for assessing blueberry internal bruising using deep learning models, which can support the development of blueberry cultivars tailored for machine harvesting.

## Data Availability

The raw data supporting the conclusions of this article will be made available by the authors, without undue reservation.
